# Nanoparticles: Synthesis and Their Role as Potential Drug Candidates for the Treatment of Parasitic Diseases

**DOI:** 10.3390/life12050750

**Published:** 2022-05-18

**Authors:** Hammad Ur Rehman Bajwa, Muhammad Kasib Khan, Zaheer Abbas, Roshan Riaz, Tauseef ur Rehman, Rao Zahid Abbas, Muhammad Tahir Aleem, Asghar Abbas, Mashal M. Almutairi, Fahdah Ayed Alshammari, Yasser Alraey, Abdulaziz Alouffi

**Affiliations:** 1 Department of Parasitology, Ankara University, Ankara 06100, Turkey; hrbajwa22@gmail.com; 2 Department of Parasitology, University of Agriculture, Faisalabad 38040, Pakistan; kasibdadra@hotmail.com (M.K.K.); dr_zaheer97@yahoo.com (Z.A.); raouaf@hotmail.com (R.Z.A.); 3 Department of Animal Nutrition and Nutritional Diseases, Ankara University, Ankara 06100, Turkey; roshansahil04@gmail.com; 4 Department of Parasitology, The Islamia University of Bahawalpur, Bahawalpur 63100, Pakistan; 5 MOE Joint International Research Laboratory of Animal Health and Food Safety, College of Veterinary Medicine, Nanjing Agricultural University, Nanjing 210095, China; dr.tahir1990@gmail.com; 6 Faculty of Veterinary and Animal Sciences, MNS-University of Agriculture Multan, Multan 60650, Pakistan; abbasasghar255@gmail.com; 7 Department of Pharmacology and Toxicology, College of Pharmacy, King Saud University, P.O. Box 2457, Riyadh 11451, Saudi Arabia; mmalmutairi@ksu.edu.sa; 8 College of Sciences and Literature Microbiology, Arar Northern Border University, Arar 73211, Saudi Arabia; fahdah.ayed@nbu.edu.sa; 9 Department of Clinical Laboratory Sciences, Central Research Laboratory, College of Applied Medical Sciences, King Khalid University, Abha 62217, Saudi Arabia; yahamd@kku.edu.sa; 10 King Abdulaziz City for Science and Technology, Riyadh 12354, Saudi Arabia

**Keywords:** nanoparticles, mechanisms of action, synthesis, parasite control

## Abstract

Protozoa, helminths and ectoparasites are the major groups of parasites distributed worldwide. Currently, these parasites are treated with chemotherapeutic antiprotozoal drugs, anti-helminthic and anti-ectoparasitic agents, but, with the passage of time, resistance to these drugs has developed due to overuse. In this scenario, nanoparticles are proving to be a major breakthrough in the treatment and control of parasitic diseases. In the last decade, there has been enormous development in the field of nanomedicine for parasitic control. Gold and silver nanoparticles have shown promising results in the treatments of various types of parasitic infections. These nanoparticles are synthesized through the use of various conventional and molecular technologies and have shown great efficacy. They work in different ways, that include damaging the parasite membrane, DNA (Deoxyribonucleic acid) disruption, protein synthesis inhibition and free-radical formation. These agents are effective against intracellular parasites as well. Other nanoparticles, such as iron, nickel, zinc and platinum, have also shown good results in the treatment and control of parasitic infections. It is hoped that this research subject will become the future of modern drug development. This review summarizes the methods that are used to synthesize nanoparticles and their possible mechanisms of action against parasites.

## 1. Introduction

**Figure 1:**
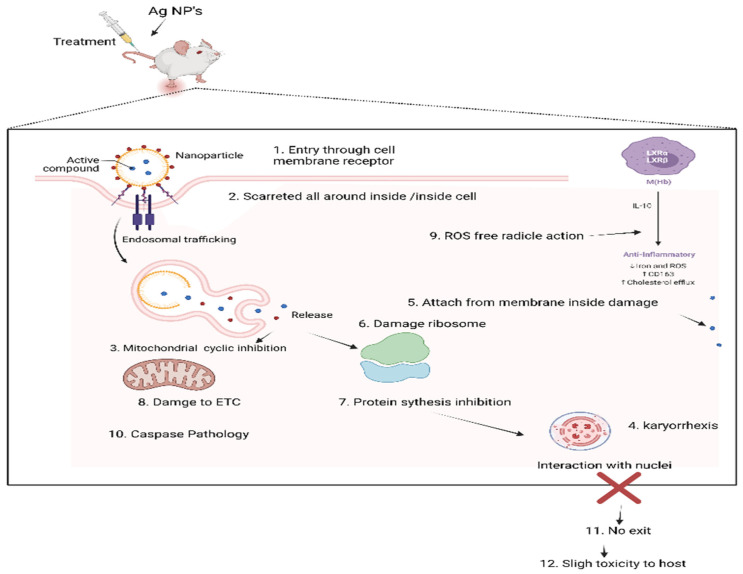
General mechanisms of action of nanoparticles against parasites.

Parasites are organisms that live on or within other organisms and utilize the resources of their hosts in terms of food, shelter and safety [1]. These parasites are classified into three groups: protozoa, helminths and ectoparasites. The classifications are based on the parasites’ morphology, genetic variation, evolution and adaptations [2]. Their transmission occurs through the fecal-oral route, vectors, or direct contact [3]. These parasitic infections are highly prevalent, because of a lack of suitable treatment options and a lack of research in pharmaceutical sciences [2]. Most of these diseases are considered to be neglected tropical diseases, and the death toll from such parasitic infections is high. These parasitic infections have been treated customarily by chemotherapeutic agents and ethnobotanicals [3]. The chemotherapeutics are antiprotozoal, antihelminthic, insecticide and acaricide agents. Among antiprotozoal drugs, antimalarials, antiamoebic [4], antigiardial, trypanocidal, antileishmanial and anti-toxoplasmic agents are used to treat various protozoal diseases [5]. These agents act in a variety of ways: they disrupt the parasite DNA, inhibit protein synthesis [6], damage the parasite membrane and kill the protozoa (Figure 1). However, due to the development of resistance by these protozoa, some of these treatments are no longer effective [7].

Other options for treatment and control of the parasites include the use of ethnobotanicals or vaccines [8]. Ethnobotany is the investigation of the efficacy of plant-based materials. such as flowers, leaves, shoots and roots against particular infections [4]. There is limited research in this field, due to a lack of interest from multinational pharmaceutical companies, which forecast low economic returns on treatments for diseases of high prevalence in poor and underdeveloped areas [9]. This low level of interest means that only 1% of ethnobotanical drugs currently used have been discovered in the last 40 years [10]. A disadvantage of ethnobotanicals is that the chemical composition of the plants varies from plant to plant due to differences in soils and ecological areas [11,12]. The dosage of active ingredients is also not controlled. These drugs remain under consideration for development [5]. In the last few decades, there has been enormous development in the field of vaccines to control these diseases. However, most vaccine development is focused on pathogens that are responsible for disease outbreaks around the world [1]. Vaccine development against parasites is difficult, due to immense antigenic variation during the immunogenic response. During the first exposure of a vaccinated subject to the parasite [13], the parasite is targeted by the immune system; after this, the parasite re-attacks the host with antigenic shift or antigenic mimicry. The parasites have developed various mechanisms that help them to escape the immune system. Hence this area is also understudied.

In the last decade, scientists have found that nanoparticles (NPs) can be used to treat bacterial, viral, fungal and parasitic diseases [14]. Certain materials, when reduced to nano size, undergo a change in their properties. These nano-sized structures have been used extensively for the monitoring, diagnosis, treatment and control of infectious diseases [15]. These NPs have well-defined chemical, mechanical and optical properties. One of the properties of these NPs is their cytotoxic effect, which varies with size, form, charge, purity and stability of the NPs [1]. They have shown excellent results in the treatment of parasitic infections [16].

## 2. Nanoparticle Development/Synthesis

Various methods of synthesis of NPs have been developed based on the size required, shape, or composition of the materials [17]. The properties of the materials greatly influence all these parameters. These NPs are synthesized or prepared through the use of in-vitro or conventional techniques. Some of these methods are explained below [18].

### 2.1. Photochemical and Thermal Disintegration

Photochemical and thermal disintegration involves the decomposition of NP precursors at high temperatures in boiling solvents [19]. However, it is very difficult to attain the unstable NP phase from the reactive phase because of the high temperature [17]. The process is endothermic because high temperatures are required to break the bonds of the starting materials. Photochemical analysis is used to isolate NPs of known sizes and to study the composition of the nanomaterials [20]. This photochemical and thermal disintegration technique has been widely used for nanoparticle synthesis. Advantages of this technique include the fact that it is simple and inexpensive, there is irradiation control, it is carried out at room temperature, it does not require lots of skill to perform and it is ecofriendly. There are some disadvantages, including sensitive and small sized nanoparticles and surface damage. This technique is still under development [21].

### 2.2. Bimetallic Nanoparticle Synthesis

Bimetallic NPs are combinations of NPs with different architectures. These NPs optimize the energy of plasmon adsorption, a band of metallic solution which is a multipurpose tool for bio-sensing [22]. The properties of these bimetallic NPs may differ from those of elemental particles. They have been studied extensively in the last decade [23]. Various methods have been proposed for the characterization and preparation of bimetallic NPs [1]. Currently, nanotechnologists are focusing on the preparation of new bimetallic NPs that offer different features, such as alloys, core-shelled and contact-aggregated NPs and many other forms [24]. It has been observed that, through bi-metallization, the catalytic properties of NPs can be improved to an extent that cannot be achieved with the use of monometallic catalysts [25]. Electronic effects, or the charge transferring ability, of bimetallic catalysts, play a major role in their action. Alloying of the elemental particles that are used for bi-metallization results in a change of structure of the bimetallic NPs [24], such that the degree of freedom of the bimetallic NPs is greater than that of the monometallic starting materials [25]. The catalytic activities of various bimetallic NPs have been compared. Researchers have developed and correlated various methods of preparation through physical and spectroscopic analysis [26]. The miscibilities and structures of bimetallic NPs are dependent upon the preparation conditions of the two metals [24]. Generally, these bimetallic NPs are synthesized by concurrent reduction of two metal ions. Then they are stabilized through the use of molecular strategies, such as steric hindrance or static-electronic repulsion [27]. The properties of the particle that is produced between the core-shell and the homogeneous alloy depend on the reduction conditions that can be attained [28]. Therefore, control of the rate of reduction of the components leads to controlled production of NPs of the required size, structure and shape. Bimetallic nanoparticle synthesis is an advanced method for nanoparticle synthesis. It has various advantages. The main advantages include two metals are incorporated in a single nano-entity. This method is more green; mainly plant-based sources are involved, so it is ecofriendly, simpler and has an easy delivery system [21]. The disadvantages are the high cost of equipment, the continuous observation of crystal phase and poor stability. This method has been applied on few metallic crystals. So, more research is required to make it more efficient [29].

### 2.3. Chemical and Electrochemical Reduction

This production method involves two processes: reduction and interaction. The interaction takes place between the polymeric and metallic species [30]. Reducing agents that are used in this process include sodium borohydride, Tollen’s reagent, hydrogen and ascorbate [31]. This method is most commonly used in the synthesis of silver NPs (AgNPs), which have shown promising results in parasite control [32]. Successive reduction reactions are used to synthesize bimetallic NPs that exhibit a core-shell structure [20]. The structure of one metal element is broken open over synthesized monometallic NPs of other metals [25]. Recently this method has been modified to become electrochemical reduction, in which electricity is used as the driving force that controls the whole process [33]. Electric current passes between two electrodes due to the presence of electrolytes. This method is used to synthesize metallic NPs [17]. The dissolved, metal-based anode sheet and metal-based salt are reduced to metallic particles at the cathode. Metal-based NPs are unstable during synthesis and must be stabilized with the addition of tetraalkylammonium salts [34]. Compared with chemical reduction, this is a low-cost method of manufacture that produces pure NPs. Moreover, this technique has certain merits, including cost effectiveness, high purity, optimized particle size and controlled particle sizing. The demerit of this technique is its complexity and the fact it requires skilled personnel [35].

### 2.4. Sol-Gel Technique

The sol in this technique is a colloid-based solid particle suspension in a liquid. The sol contains sufficient liquid for the generation of Van der Waal’s forces [36]. In contrast, the gel comprises more solid than liquid and is a semi-rigid mass, in which ions and particles form a stable mesh. In most gel systems, there are high numbers of covalent bonds [36]. When sol and gel are combined, hydrolysis and condensation take place. Bimetallic NPs, in particular, are prepared through the use of the sol-gel technique. This method is cost-effective and simple and the NPs that are obtained in this way are of good quality [36]. The chemical composition of the product can be controlled because the sol-gel method is used at low temperatures. The use of the method has been hampered due to a scarcity of funding [37], the need for specialist equipment and concerns regarding the toxicity of the NPs produced. However, a cost-effective and simple method that requires minimal apparatus is explained below [38]. This technique is quite useful because of its advantages, including simple, excellent quality NPs, economic factors, the fact that it requires low temperature and the product’s chemical composition can be controlled. The major limitations of this technique are the high cost of materials to be used and excessive shrinkage of volume of organics during the drying process [35].

### 2.5. Green Synthesis

Synthesis of NPs through the use of various plant extracts offers a method that is cheaper, less toxic and simpler to set up than the methods described above [39]. Different plant species that are used for NP synthesis are listed in Table 1. These plants contain active ingredients that have antibacterial, antiviral and antiparasitic properties [40]. Extracts are prepared from leaves, roots, shoots, stems or bark. The active ingredients in these parts reduce the metals in NPs by acting as reducing agents. The NPs that are produced must be stored in specific conditions; [41] for instance, gold NPs (AuNPs) are stored in the dark to avoid oxidation. This synthesis technique has one drawback, which is that the NPs that are produced are of uneven size [42]. This technique is in development to improve it, but NPs that have been synthesized through the use of this technique have been reported to work well and this method shows promise in the development of modern drugs to treat parasitic diseases [43]. Currently, this technique is preferred, because of its safety towards the environment. Other main advantages include the fact it is simple, cost effective, requires less materials, uses less chemicals, is more ecofriendly and consumes less energy [44]. Furthermore, it has few disadvantages; such as less purity, uncontrolled product size and less knowledge of botanicals [45].

## 3. Metal-Based Nanoparticles and Their Antiparasitic Significance

**Figure 2:**
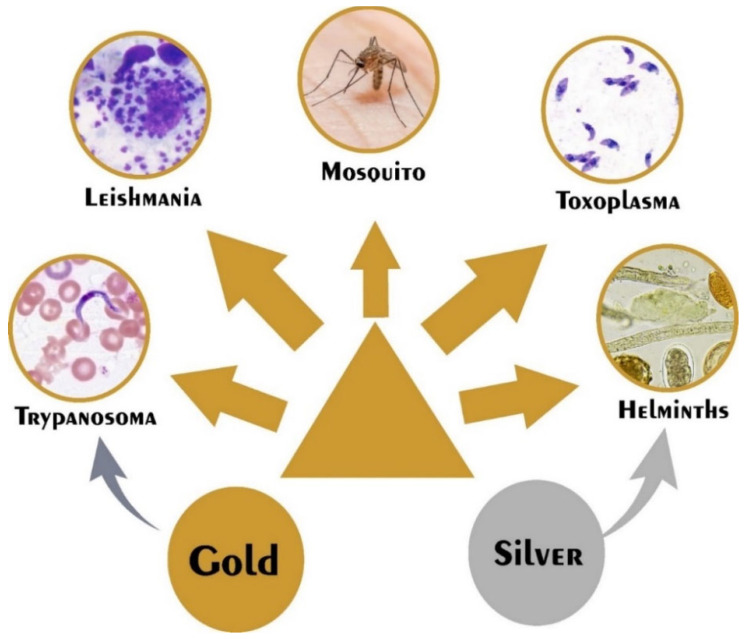
The antiparasitic spectrum of action of silver and gold nanoparticles.

A variety of metals are employed in NP synthesis. Those that are of antiparasitic significance are shown below (Figure 2).

### 3.1. Aurum (Gold)-Based Nanoparticles

AuNPs show potential in the treatment of various disease conditions [49]. AuNPs occur in different forms, particularly two oxidized forms (Au^+1^, Au^+3^) and one non-oxidized form (Au^0^) [33]. Oxidized forms are reduced to non-oxidized forms through the use of chloroauric acid as a precursor. AuNPs are prepared by use of either in-vitro techniques or conventional methods. After preparation, the fresh batches are stored in the dark to avoid their oxidation by light [50]. The effectiveness of AuNPs has been shown against various parasites of public health importance, including the protozoa [51] *Toxoplasma gondii*, *Trypanosoma* spp., *Leishmania* spp. [52] (*L. tropica*, *L. donovani*) and *Cryptosporidium* spp. (*C. parvum*); helminths including trematodes (*Schistosoma* spp.) [48] and cestodes (*Raillietina* spp.); and vectors including mosquito, genus Aedes (spp. *A. aegypti* L.), [26] Anopheles (spp. *A. stephensi*), and Culex (spp. *C. quinquefasciatus*); and dipteran flies. Currently, AuNPs are considered novel products in the field of drug discovery [1].

### 3.2. Aurum-Based Nanoparticles for Vector Control

Mosquitoes serve as a vector for dengue fever, yellow fever, Zika virus, Mayaro virus and many other zoonotic diseases [53]. AuNPs, synthesized through the use of the Cymbopogon citrus leaf extract, have shown good efficacy against different species of mosquito, including Aedes (*A. aegypti*) and Anopheles (*A. stephensi*), according to measures of LC50 (effective concentration that kills 50 percent of a population of mosquitoes) [43]. In contrast, in a study, the synthesis of AuNPs from the *Couroupita guianensis* flower extract showed higher toxicity against the Anopheles mosquito than AuNPs synthesized from other starting materials [33]. In another study, AuNPs coated with zein (a protein-based coating) showed much higher activity against different species of mosquito compared with the NPs that were produced in the former studies. On microscopic examination of the mosquitoes [54], it was reported that the internal organs of the vector had disintegrated and hairs had been removed from the caudal region of the body.

### 3.3. Aurum-Based Nanoparticles for Control of Protozoa

Protozoan parasites are responsible for various diseases. They are notoriously difficult to control. Success has been achieved with the use of AuNPs against these parasites. Some of these protozoans are described below [9].

#### 3.3.1. Plasmodium

Plasmodium is an intracellular parasite that is transmitted through the Anopheles mosquito and causes malaria in infected individuals [55]. Partial success has been achieved in the fight against malaria by controlling the vector through the application of insecticides, but the development of resistance in the parasite to insecticides has worsened the situation. Treatment of infected individuals with AuNPs gave good results [54]. In one study, patients were treated with AuNPs that had been synthesized from *C. guianensis* flower extract. Tremendous antimalarial properties were observed. Similarly, in another study, mice were experimentally infected with *Plasmodium berghei* and treated with Streptomyces LK-3-mediated AuNPs [56]. The findings suggested that these NPs could be considered potential antimalarial drug candidates. AuNPs have also shown good results in the diagnosis of malaria. Recently, a cost-effective diagnostic technique was developed that was inspired by the “coffee-ring” phenomenon; the occurrence of histidine-enriched protein-II, which is a biomarker of *Plasmodium falciparum* [57]. This protein was detected by the use of a surface-coupled ring of nitrilotriacetic acid aurum-plated polystyrene micro-particles in infected individuals. This kit, based on AuNPs, indicates the presence of the protein by the formation of a ring that can be analyzed visually. Hence, this kit can be used to detect malarial parasites [58].

#### 3.3.2. Leishmania

Leishmaniasis is a vector-borne parasitic disease that is one of the most fatal worldwide and is highly prevalent in tropical areas. Transmission occurs through the sandfly vector. About seven million cases are reported annually [59]. The prevalence of *Leishmania* is high because there is a lack of suitable treatment options. Antileishmanial pharmaceutical drugs that are used against infection are expensive and not widely available [60]. Chemotherapeutic agents have shown tremendous results targeting leishmaniasis in recent decades; they are indicated in Table 2. However, these chemotherapeutic agents have certain limitations; [61] (1) Severe side effects in parenteral use; (2) Treatment failure and drug resistance; (3) unequal distribution of drug at target site; (4) lack of research in herbal medicine. In later studies it was revealed that *Leishmania* sp. have developed resistance against these chemotherapeutic agents. Further studies were conducted in which genomic studies of leishmania for drug development were carried out [62]. It was revealed that *Leishmania* sp. modified its genes that were targeted by these drugs. The major issue was unequal distribution/reach of drug at the target site, because of the intracellular nature of the parasite. Then, another milestone was achieved by the use of nanomaterials as drug delivery vehicles to target *Leishmania* sp. In some studies, these nanomaterials have shown greater efficacy and controlled the release of the drug at the target site. As well as this, there was less toxicity [63]. Currently, this area of drug development is still understudied. It will take more time to establish more suitable nanomaterial-based chemotherapeutic agents that may have no resistance [63]. In recent years, NP treatment of Leishmaniasis has shown successful results in the diagnosis and treatment of the infection. In a study, promastigotes of *Leishmania brasiliensis* were targeted in skin lesions with AuNPs that were incorporated into membranes of natural rubber [52]. The results showed a decreased growth rate of promastigotes, a change in the physiological behavior of the promastigotes and decreased lifespan [59]. These particles also aided in the angiogenesis of infected areas of skin. There was no issue of resistance. Treatment with AuNPs, coupled with microwave radiation at a frequency of 2450 MHz, showed greater efficacy of the particles, as compared with the above study [16]. Similarly, another cheap method was used to target different *Leishmania* species. AuNPs were fabricated with *S. cuneata* and Wills phytochemicals. The antileishmanial activity was greater than that measured in the above studies.

Various NP-based diagnostic techniques are used, including nano-sensors, to detect Leishmania infection [64]. Furthermore, a diagnostic kit has been developed in which AuNPs are conjugated with four oligonucleotide probes that target Leishmania DNA. A color change from red (the medium) to purple indicates the presence of leishmanial DNA. The minimum detection limit is 11.5 ng/µL of the sample [52]. This technique has shown good results in the detection of canine leishmaniasis. It is suggested that AuNP-based treatments and diagnostic tools have provided fresh impetus to drug development.

#### 3.3.3. *Toxoplasma gondii*

Toxoplasmosis is caused by the intracellular protozoan parasite *Toxoplasma gondii*. The infection is usually asymptomatic but can be life-threatening in immunocompromised individuals [68]. Recently, *Toxoplasma gondii* was treated with NPs. In-vitro studies were conducted in which *Toxoplasma gondii* tachyzoites were targeted with different concentrations of AuNPs: 100–1000 ppm, to which the parasites were exposed for time periods of between 30 and 180 min [69]. The concentration of AuNPs and period of treatment were found to be directly proportional to the number of *Toxoplasma gondii* tachyzoites that were killed. The results revealed that AuNPs offered potential as drugs for the treatment of Toxoplasmosis [70].

In another study, AuNPs conjugated with antibodies against *Toxoplasma gondii* showed greater efficacy in the treatment of Toxoplasmosis than the antibodies alone [49]. In the same study, the researchers developed a diagnostic test, in which the agglutination reaction between antigen-coated AuNPs and *Toxoplasma gondii* antibodies was detected and the reaction was observed through use of a piezoelectric device. This technique was found to be reliable for the detection of *Toxoplasma gondii* infection [71].

#### 3.3.4. Trypanosoma

African trypanosomiasis is a neglected tropical disease caused by *Trypanosoma* (*T.*) *brucei gambiense* or *T. brucei rhodesiense* [72]. The tsetse fly transmits the infectious agent. In Latin America, the infection is caused by *T. cruzi* and *Triatoma bugs* transmit the parasite, which causes a disease commonly known as Chagas disease [73]. Currently, there are no reliable treatment options for the control of this disease. Recently, treatment of trypanosomiasis with NPs has shown promising results. In a study, various *Trypanosoma* spp. were targeted by inorganic metal NPs. AuNPs and AgNPs were used against *T. brucei gambience*, *T. cruzi*, *T. evansi* and *T. congolense* [74,75]. These NPs showed great efficacy against *T. brucei gambience* and *T. cruzi* and growth was arrested by 50% in studies that targeted *T. evansi* and *T. congolense*. Similarly, in another study, arginine kinase was targeted through treatment with AuNPs and AgNPs. Arginine kinase (phosphotransferase) is essential for *Trypanosoma* energy metabolism [73]. These NPs also showed effective arrest of parasite growth. AuNPs were also used in the diagnosis of Trypanosoma with the use of confocal length microscopy at a wavelength of 633 nm [74]. Endocytosis was observed in epimastigotes of *T. cruzi.* Epimastigotes ingested the AuNPs and the formation of gold ring complexes was observed. It was concluded that, in theory, this could be a reliable technique for the diagnosis of Trypanosomiasis [74].

### 3.4. Silver (Ag)-Based Nanoparticles

AgNPs have many applications in the field of biomedical sciences. They have been used extensively in pharmaceuticals, bioimaging, biomedical devices and as antipathogenic agents [76]. Recently, scientists discovered their benefits in the control of parasites. AgNPs are synthesized through the application of various in-vitro techniques, such as electrochemical reduction, the sol-gel technique and green synthesis [26,36]. Green synthesis is favored because chemical methods are expensive and the reagents used for electrochemical reduction are toxic. Plant extracts, such as *Solanum trilobatum*, *Sechium edule* and the fruit of the Ficus tree, are used in green synthesis [77]. These show promise in the treatment of parasitic infections, but the NPs are produced in uneven sizes of 10–250 nm. The antiparasitic impact of AgNPs is indicated below.

### 3.5. Ag-Based Nanoparticles for Vector Control

Mosquitoes serve as vectors for various parasitic diseases. Parasitologists have applied various chemical and biological methods for their control [78]. However, due to immense genetic variation and the development of resistance in mosquitoes, these techniques are no longer effective [55]. In the last decade, parasitologists have reported excellent results in mosquito control through the use of green synthesized AgNPs. In one study, AgNPs synthesized from *Mimosa pudica* leaf extract were used against *Anopheles subpictus* and *C. quinquefasciatus* to good effect [26]. When AgNPs were synthesized through the use of methanol with *Mimosa pudica* leaf extract, the efficacy of the AgNPs was increased [79]. In another study, AgNPs produced from *Euphorbia hirta* leaf extract were tested against *A. stephensi* and the highest LC_50_ and LC_90_ data that were reported were 27.98 ppm and 69.94 ppm, respectively. These NPs showed excellent larvicidal activity [40]. AgNPs synthesized from the fungal species *Chrysosporium keratinophilum* and *Verticillium lecanii* showed good activity against the pupae and larvae of *A. stephensi*, *C. quinquefasciatus* and *A. aegypti* [80]. These studies taken together show that these green synthesized AgNPs can be used as alternatives to other treatment and control options for mosquitoes.

### 3.6. Silver (Ag-Based) Nanoparticles for Control of Protozoa

#### 3.6.1. Plasmodium

AgNPs, as with AuNPs, are effective in the treatment and control of malaria caused by plasmodium. In one study, AgNPs were synthesized by the green method from *Azadirachta indica* and *Ocimum sanctum* leaf extracts [80]. The size of the nanoparticles was estimated through the use of electron microscopy (Transmission electron microscopy) and the dynamic light scattering method or DLS method. Furthermore, studies revealed that the DLS method was more accurate in size measurement, as compared to TEM (Transmission electron microscopy) [81]. The anti-plasmodial activity of these AgNPs was found to be good against *Plasmodium falciparum* [56]. In another study, AgNPs that were synthesized from plant extract and tested against *P. falciparum*, showed dose-dependent anti-plasmodial activity. The results showed that the activity of these AgNPs was considerably higher than that found in a previous study. This approach remains under development [26].

#### 3.6.2. Leishmania

In-vitro studies of AgNP treatment against *Leishmania tropica* reported the formation of reactive oxygen species (ROS), to which Leishmania is known to be highly sensitive [82]. In this study, the morphological features and proliferation, infectivity and survival rates of Leishmania were studied in the presence of AgNPs. AgNPs were found to impair the metabolism, decrease the survival rates and inhibit the proliferation of *Leishmania tropica* promastigotes [59]. Moreover, the reactivity of AgNPs was enhanced by application of UV light, which increased their impact by two- to 6.5-fold [83,84]. In another study, the effect of AgNPs was enhanced through the use of both infrared and UV light. ROS were formed, which led to the development of toxicity in Leishmania and resulted in the killing of the parasite [26]. In another study, AgNPs, prepared from *Anethum graveolens* leaf extract, were structurally analyzed through the use of X-ray diffraction and their size (35 nm) was estimated through the use of transmission electron microscopy. The combination of AgNP with Miltefosine (12 µM) resulted in the death of amastigotes and promastigotes of *Leishmania* [76]. The multiple ways in which NPs act mean that they can be used alone or in combination with other agents. This may be a reliable form of treatment in the near future.

#### 3.6.3. *Toxoplasma gondii*

*Toxoplasma gondii* is a coccidian protozoan parasite that is prevalent across the globe [85]. Various regimes can be used for its treatment and control. However, due to vaccine failure and resistance to chemotherapeutic agents, most of these options are not reliable [49]. Therefore, scientists are testing NPs against *Toxoplasma gondii.* In one study, the application of silver, gold and platinum NPs against *Toxoplasma gondii* resulted in damage to the mitochondrial membrane of the parasite and 90% inhibition of infection [28]. There was no effect on host cells. In another study, AgNPs were combined with Chitosan and changes that were observed in the *Toxoplasma gondii* parasite included tachyzoite shape deformity, movement arrest and a decrease in the parasite burden [42]. The gamma interferon number was also increased in treatment groups. Moreover, in in-vitro studies, biogenic AgNPs were tested against *Toxoplasma gondii* in HeLA cell lines at concentrations that ranged from 0.25 μM to 12 μM. Parasite proliferation rates were significantly reduced [86]. Furthermore, AgNPs that were prepared from *Phoenix dactylifera* and *Ziziphus christi* extract were used against *Toxoplasma gondii* and they showed improvement in serum profile and reduced immunoreactivity of infected mice. These studies show that AgNPs can offer a reliable treatment regime for the control of Toxoplasmosis [87].

## 4. Nanoparticle Toxicity and Safety

Parasites are considered more pathogenic to animals and humans than bacteria because they cause chronic diseases [17]. They may live for years in the environment and their hosts due to their complex life cycle stages [88]. Each developmental stage produces a distinct sensibility against the same drug [89]. Additionally, the bioavailability of antiparasitic drugs is very low due to insolubility and short half-life. For example, important antiparasitic drugs like ivermectin and praziquantel are more prone to enzymatic degradation and poor penetration across the biological membrane of cells. This results in reduced bioavailability and the expected therapeutic effect of the drug is not achieved [90]. There is an immense problem that is faced by physicians and medical experts while treating these parasitic infections [91]. The problem is drug resistance due to the blind use of these chemotherapeutic agents in the field. These chemotherapeutic agents include antibiotics, antifungals, antiprotozoal and antihelmintics [92]. Various studies have revealed that the continuous use of these chemotherapeutic agents results in modified pathogens that no longer respond to older medicine regimes. So, nanomedicine has paved the way for effective antiparasitic therapy [93]. The problems create hindrance in the effective control of parasitic diseases. In this scenario, targeted delivery of antiparasitic drugs is a major concern which is only possible with nanoparticles [94]. Different nanocarriers, in the form of nanocrystals, solid lipid nanoparticles (SLNs) and liposomes have been introduced that can be administered through oral, intestinal, intravenous, and pulmonary routes. These nanocarriers provide physical stability and targeted and controlled release of drugs [95,96,97,98,99,100]. Furthermore, they protect the loaded drug from degradation by enzymes [98], and it has been observed that the bioavailability and mean residence time of praziquantel was increased by 5.67 and 4.94 folds with hydrogenated castor oil SLNs. NPs are metabolized easily into the liver and change physicochemical properties through enzymatic degradation. These nanoparticles can be metabolized by endocytosis facilitated by the reticuloendothelial system. Ref. [99] observed that the amphotericin B liposome can fuse into the cell and counter intracellular parasites. In the cell, NPs can be hydrolyzed with the help of lysosomes and release the drug to work against intracellular parasites. Besides the metabolism of NPs in the liver, they can be excreted by the glomerular excretion system of the kidneys. However, the excretion of these NPs mainly depends on their size, shape, and charge. It has been observed that NPs with smaller sizes (80–120 nm) can be excreted by the kidneys easily, while larger particles undergo hepatobiliary excretion. Furthermore, the excretion of NPs also depends on the charge. Regarding [100], it was observed that the NPs with higher charge (+34.4 mV) were excreted through feces, bypassing the gastrointestinal tract; however, the particles with lesser charge (−17.6 mV) remained isolated in the liver. Thus, the safety and efficacy of NPs greatly rely on their physicochemical properties [101]. To initiate the application of these nanoparticles, it is not only really necessary to ensure that the nanoparticles have the rate of absorption [102], sustained release, and effective intracellular delivery at the appropriate concentration for the expected period of time to produce satisfactory therapeutic outcomes, but also, they should be safe, cheaper, and easily reproducible at larger scales [103]. Currently, studies regarding the safety of these nanoparticles are short because of less systemic study and research [104]. The core concept of nanomedicine is the careful designing of nanoparticles to use fewer drugs with lower concentrations to achieve more effective results. Although studies have reported that most of the nanoparticles are safe in use and may be able reduce the adverse effects of various drugs [105], there are some studies that state that some nanoparticles have toxicity issues. There should be more attention to the discovery and synthesis of novel biofriendly nanomaterials with no toxicity [105]. The physical and chemical structure of these nanoparticles should have excellent physiological and biological compatibility. They should be non-toxic, nonimmune-toxic, and be able to be completely excluded from the body in a reasonable time [106]. After preparation into nanoparticles, their safety should be evaluated using proper biosafety evaluation systems at every level, including molecule, cell, tissue, organ, and organismic levels [107].

## 5. Conclusions and Future Prospects

Major outbreaks of disease occur around the world due to neglected tropical parasites. Parasites have been treated through a variety of methods for 50 years but these methods have become ineffective. Overuse of chemotherapeutic agents and some contraindications to chemotherapeutic agents have limited their use. Secondary infections can sometimes damage host physiology and parasites have developed immunity, thus indicating a need for other options. Ethnobotanicals have shown promise but lack funding and are of limited interest to investors, which means that only 1% of ethnobotanicals have been developed in the last 50 years. Therefore, a lack of knowledge regarding dosage information inhibits their use to treat parasites. The alternative option for ethnobotanicals is vaccine development; however, due to homogeneous antigenic variation, this task is challenging in parasitology. Parasites develop mechanisms to escape the host immune system. Hence, parasitologists have studied the use of NPs to control parasites. The unique properties of NPs offer advantages over other treatment options, such as their lack of toxicity and zero resistance from parasites. Most of the work performed to date regarding parasite control by NPs has been performed against protozoa such as Plasmodium, Trypanosoma and Leishmania. NPs prepared through different conventional and molecular methods have been shown to kill the parasite or arrest growth and they can be used to diagnose parasitic diseases. Gold and silver NPs have undergone the most studies, but other NPs, such as iron, nickel, platinum and copper, remain understudied for their efficacious use in disease control of animals and humans. Nanotechnology is the most recent technique in medical and pharmaceutical industry. There has been enormous development in medical and pharmaceutical industries in recent decades regarding the use of nanotools for treatment and control of infectious diseases. However, there is a need for more research to understand the nanoparticle mechanisms of action for development of safe diagnostic and treatment options. Nanoparticles are likely to offer a major breakthrough in the field of parasite treatment and control.

## Figures and Tables

**Table 1:** Mechanisms of action and therapeutic outcomes of nanoparticles synthesized from herbal sources.

Nanoparticles	Source	Common Name	Mechanism of Action	Infection	Therapeutic Outcome	References
Silver (AgNPs)	*Solanum trilobatum* and *Mimosa pudica*	Pea eggplant and shame-plant	Interferes with cell membrane, and damages DNA and electron transport	Malaria, Leishmaniasis, Helminth infections	Inhibition of the growth of *Plasmodium* (*P.*) *falciparum* Inhibition of proliferation and metabolic activity of promastigotes. Good antileishmanial activity in vitro and in vivo Enhanced anti-helminthic activity against worms	[31,32]
Some metal oxides (MO) (Fe_3_O_4_, MgO, ZrO_2_, Al_2_O_3_ and CeO_2_)	*Lawsonia inermis*, *Azadirachta indica*, *Camellia sinensis*, and *Cinnamon zeylanicum*	Henna tree, neem, tea plant and cinnamon tree	Disrupts the cell membrane, accumulates inside the cell and produces toxic H_2_O_2_ Damages cell membranes and releases reactive oxygen species	Malaria, Leishmaniasis	Enhanced cytotoxic effects on promastigotes of *Leishmania major* via induction of apoptosis	[42,46,47]
Gold (AuNPs)	*Acalypha indica*	Indian mercury plant	Heavy electrostatic attraction, accumulation at cell surfaces, and interaction with cell membrane	Malaria, Helminth infections	Moderate delayed parasitemia rise in vivo, moderate anti-plasmodial activity against *P. falciparum* Affects physiological functioning of parasite by causing paralysis and leading to death	[48]
Zinc oxide NPs	*Passiflora caerulea*	Blue passionflower	Disrupts the cell membrane, accumulates inside the cell and produces toxic H_2_O_2_	Helminth infections	Inhibits adenosine triphosphate production and the contractile movement of the parasite	[42,47]
Iron oxide NPs	*Gardenia jasminoides* and *Azadirachta indica*	Cape jasmine and neem	Cytotoxicity (by producing reactive oxygen)	Helminth infections	Induces oxidative stress	[8,46]

**Table 2:** Chemotherapeutic treatment options for various types of Leishmaniasis.

Type of Leishmaniasis	*Leishmania* sp.	Status of Chemotherapeutic Agent	Drugs	Drug Resistance	Reference
Visceral Leishmaniasis	*L. donovani* *L. infantum*	Drugs of choice	Sodium stibogluconate Amphotericin B Pentamidine	Yes	[65,66,67]
Cutaneous Leishmaniasis	*L. major*, *L. tropica*	Drugs of choice			
Mu-cutaneous Leishmaniasis	*L. donovani* *L. major*				

## Data Availability

The data presented in this study are available within the article.
